# Left knee septic monoarthritis in a pediatric patient due to shewanella putrefaciens: case report and literature review

**DOI:** 10.1186/s12941-024-00702-6

**Published:** 2024-05-10

**Authors:** Nathalie Yepes Madrid, Luis Fernando Mejia, José Fernando Gomez Urrego

**Affiliations:** 1 Pediatric specialty resident, Universidad Libre Sectional Cali, Valle del Cauca, Colombia; 2 Fundación Clínica Infantil Club Noel, Pediatric Specialty Program, Universidad Libre Sectional Cali, Valle del Cauca, Colombia; 3 Fundación Clínica Infantil Club Noel, Postgraduate coordinator of the Specialty Program, Universidad Libre Sectional Cali, Epidemiologist, Valle del Cauca, Colombia; 4 Pediatrics Specialty Research Group (GRINPED), Valle del Cauca, Colombia

**Keywords:** Septic arthritis, *Shewanella putrefaciens*, Pediatrics, Colombia, Penetrating wound

## Abstract

**Background:**

*Shewanella putrefaciens* is a gram-negative, nonfermenting, oxidase-positive, hydrogen sulfide-producing bacillus and a halophilic bacterium, known for causing unusual infections in humans and often regarded as an opportunistic pathogen. Its diverse symptoms have a significant impact on human health, with 260 documented disorders reported in the literature over the last 40 years, highlighting its potential danger.

**Case presentation:**

We present the case of a previously healthy 15-year-old male patient who sustained a self-inflicted sharp-object injury while working in the field, resulting in secondary septic monoarthritis due to *Shewanella putrefaciens*.

**Conclusions:**

This case highlights the bacteriological and clinical characteristics, as well as the antibiogram, of Shewanella spp. Given the recent increase in notifications of Shewanella infections, predominantly by *S. algae* and *S. putrefaciens*, it is essential to consider these pathogens in patients with a history of contact with bodies of water. Special attention must be paid to their resistance patterns in patient management to prevent the development of intrinsic antimicrobial resistance.

## Background

The discovery of bacteria belonging to the genus *Shewanella* dates to 1931, with their first mention in medical literature occurring in 1973. Since then, human infections caused by members of the genus *Shewanella* have been rare, primarily reported in regions with warm climates. Although initially regarded as part of mixed bacterial flora, after their discovery in humans, several cases of monomicrobial infections have been documented, highlighting the pathogenic potential of *Shewanella spp*. This has led to an increase in the reporting of cases involving this bacterial species in countries with temperate climates [1, 2].

*Shewanella spp.* are gram-negative bacteria that are rarely pathogenic, typically found in marine environments but also capable of thriving in dairy products, oil, and carcasses. There are over 25 species of *Shewanella*, with only two known to be invasive towards human hosts: *Shewanella putrefaciens* and *Shewanella algae* [1].

*S. putrefaciens* is a gram-negative, nonfermenting, oxidase-positive bacterium that produces hydrogen sulfide. It is a halophilic bacterium known to play a significant role in the spoilage of frozen food products such as fish, meat, and poultry. In humans, it is responsible for unusual infections and is considered an opportunistic pathogen in many patients, particularly those with malnutrition, immunosuppression, and chronic kidney disease undergoing peritoneal dialysis. Its symptoms vary widely in the literature, indicating a significant impact on human health, with a total of 260 disorders described in the literature over the last 40 years, highlighting the potential danger it poses [1, 3–8].

*Shewanella-*related infections are reported sporadically. Hence, Keyi Yu et al. conducted a systematic review of literature published between 1978 and June 2022. Cases of Shewanella infection were identified worldwide, with a higher prevalence observed in regions characterized by warm climates, including tropical, subtropical, and temperate countries such as Australia, Belgium, Denmark, Israel, Spain, and Turkey. These infections were more prevalent in cities and coastal regions suitable for tourism and habitation, such as Taiwan, Martinique, Barbados, and the Canary Islands. The age range of affected individuals spanned from newborns to 92 years old, with a predominance in individuals over 60 years old and a male predominance of 61.7%. Among these cases, 35.16% were infected by S. algae, 28.94% by *S. putrefaciens*, and 0.7% by *S. xiamenensis*. The bacteria were mainly isolated from blood and exudates from skin lesions. While no cases were reported in Colombia in this study, a review of the literature revealed two isolated cases in Colombia, none of which occurred in the pediatric population [9, 10].

Accordingly, we present the clinical case of a pediatric patient with septic *monoarthritis* of the left knee, infected by *S. putrefaciens*, acquired in the community in its natural environment, which was initially misidentified as Pseudomonas aeruginosa.

## Case presentation

A 15-year-old male patient, native and resident of the municipality of Guapi, Cauca, Colombia, was admitted to the emergency department of his local health center with a self-inflicted sharp-object injury to his left knee, sustained accidentally while working in the fields. Initially, he underwent superficial lavage with saline solution, wound suturing, and was treated with oxacillin and clindamycin for 7 days, with no initial blood count or culture taken. Twelve days later, he began experiencing progressive pain, inability to flex or extend the knee, limited range of motion, edema, and erythema of the left knee. A comparative knee radiograph shows soft tissue edema with no evidence of bony lesions. Leukocytosis (16.69 × 103/µL) with neutrophilia (12.54 × 103/µL) without elevated C-reactive protein (0.32 mg/L) and erythrocyte sedimentation rate (13 mm/h), as stated in Table 1.

**Table 1:** Lab data

Lab data		1st day upon arrival to III level facility	10 days after broad spectrum antibiotics
CBC	White blood cells	16.69 × 10³/µL	8.52 × 10³/µL
	Neutrophils	12.54 × 10³/µL	4.41 × 10³/µL
	Lymphocytes	1.95 × 10³/µL	1.88 × 10³/µL
	Monocytes	2.00 × 10³/µL	0.85 × 10³/µL
	Hemoglobin	12.10 g/dL	10.10 g/dL
	Hematocrit	38.00%	32.70%
	Platelets	496 10³/µL	323 10³/µL
C-reactive protein		0.32 mg/L	0.1 mg/L
Erythrocyte sedimentation rate (ESR)		13.00 mm/H	10 mm/H
Blood culture		Negative	
Prothrombin time			12.70 s
Daily control prothrombin time			12.6 s
International normalized ratio (INR)			1.01
Partial thromboplastin time			31.80 s
Daily control partial time of thromboplastin (citrol)			29.7 s

He was referred to a level III facility, where the infectious diseases department doctor initiated treatment with cefepime and clindamycin. The orthopedic surgeon performed arthrotomy and debridement, revealing a wound with a moderate amount of seropurulent discharge, knee synovitis, and traumatic injury to the extensor mechanism in 70% of patients, with no evidence of distal motor deficits. A positive secretion culture for sensitive *Pseudomonas aeruginosa* was obtained, leading to the continuation of cefepime as monotherapy, as stated in Table 2.

**Table 2:** Antibiotics used during infection course

Antibiotic	Doses	Administration	Duration
Oxacillin	1000 mg every 24 h	Oral	7 days
Cephalothin	25 mg per kg per dose, every 6 h	Oral	7 days
Clindamycin	10 mg per kg per dose, every 6 h	IV	3 days
Ciprofloxacin	400 mg per kg per dose, every 12 h	IV	4 days
Cefepime	150 mg per kg per day	IV	10 days
Meropenem	2,5 mg per kg per dose, every 8 h	IV	6 days
Trimethoprim – sulfamethoxazole	4 mg per kg per dose, every 12 h	Oral	21 days

*Note*: patient’s weight was 59 kg

During a second arthrotomy debridement conducted three days after the initial procedure, the seropurulent discharge persisted, and 70% of the extensor mechanism remained damaged. Subsequent culture reports revealed *S. putrefaciens* resistant to ertapenem and cefepime. Consequently, management was guided by the antibiogram provided in Table 3, initiating treatment with ciprofloxacin. A reevaluation of the initial culture was conducted, and the microbiologist confirmed that it was indeed *S. putrefaciens*.

**Table 3:** Antibiogram

Exam	Secretion culture	
Sample site	Left Knee	
Preliminary report 1	Growth of gram-negative Bacilli	
Final report	Positive	
Microorganism	***S. putrefaciens***	
***ANTIBIOGRAM RESULTS***:		
***Antibiotic***	***Sensitive/Resistant***	MIC*
Cefepime	Resistant	> 16
Piperacillin/tazobactam	Sensitive	<=4/4
Trimethoprim/sulfamethoxazole	Sensitive	1/19
Ceftazidime	Sensitive	4
Imipenem	Sensitive	4
Amikacin	Sensitive	<=8
Gentamicin	Sensitive	<=2
Ciprofloxacin	Sensitive	0,5
Levofloxacin	Sensitive	<=1
Ceftriaxone	X	> 4

*: Minimum inhibitory concentrations were assessed according to the Clinical and Laboratory Standards Institute (CLSI) MIC breakpoints

During the third arthrotomy debridement, serosanguinous discharge was discovered without frank purulent material, alongside a persistent 70% lesion of the extensor mechanism at the distal third of the quadriceps and an osteochondral lesion on the lateral and upper aspect of the patella. A third positive culture for *S. putrefaciens*, resistant to ertapenem and cefepime, prompted an evaluation of meropenem, to which it demonstrated sensitivity upon expanding the antibiogram. Consequently, management was initiated with meropenem and trimethoprim-sulfamethoxazole.

The patient exhibited satisfactory progress with laboratory results, as shown in Table 1, and negative blood cultures. Consequently, the patient underwent the final surgical procedure for extensor mechanism reconstruction. Upon discharge, the patient was prescribed ciprofloxacin and oral trimethoprim-sulfamethoxazole for a total treatment duration of 21 days, as outlined in Table 2. Subsequent follow-up indicated an improvement in knee mobility.

## Discussion

*Shewanella spp.* are classified within the genus *Shewanellaceae*, encompassing gram-negative, nonfermenting, motile, oxidase-positive, saprophytic bacilli. This particular species is distinguished by the presence of thiosulfate reductase. Formerly known as *Pseudomonas putrefaciens*, it was initially classified in the family *Vibrionaceae* until the 1990s when it was reclassified within the genus *Shewanella*. While commonly isolated from nonhuman sources, it has infrequently been identified as a human pathogen. It is widely distributed in nature, found in environments such as natural gas, oil, forests, and fields, with its natural habitat being bodies of water, including fresh, stagnant, marine, lacustrine, fluvial, and sewage systems. Considered a colonizer or saprophyte, it thrives in damaged tissues, such as decomposing algae in the sea; thus, the microflora of the marine environment poses a potential source of human infection. The most common infections caused by marine bacteria (such as *Vibrio, Aeromonas, Plesiomonas*, and *enteric bacteria*) typically involve otitis externa, wound infections, and gastrointestinal diseases. While most marine wound infections resolve spontaneously, both *Vibrio spp*. and *Aeromonas spp*. can lead to invasive, necrotizing, and potentially life-threatening wound infections with sepsis. Hence, it is crucial to consider other marine bacteria, as observed in the case of the patient [3, 11–14].

*S. putrefaciens* was initially described and named by MacDonell and Colwell in 1985 as the sole nonfermenting gram-negative bacillus known to produce hydrogen sulfide. In 1931, Derby and Hammer isolated it from contaminated butter and classified it as *Achromobacter putrefaciens*. In 1980, Gillardy recognized three biotypes, and that same year, the United States Centers for Disease Control and Prevention (CDC) acknowledged two biotypes based on carbohydrate oxidation and growth on SS agar (*Salmonella Shigella Agar*) or nutrient agar with a high salt concentration (6.5%) [13, 15].

Throughout history, it has primarily been isolated from bodily wounds, feces, conjunctiva, urine, cerebrospinal fluid, bile, ascitic fluid, pleural fluid, and stored blood, and is largely associated with hepatobiliary disease, peripheral vascular disease, chronic leg ulcers, poor hygiene, and socioeconomic status. This is attributed to the bacteria’s ability to thrive in devitalized tissue, providing an environment conducive to opportunistic infections [2, 3, 11, 12, 14].

*S. putrefaciens* appears to opportunistically invade human hosts in a limited number of cases, making it a rare cause of bacteremia. However, soft tissue infections present with various clinical manifestations, including ulcers, gastrointestinal symptoms, cellulitis, abscesses, and wound infections, often preceded by chronic ulceration, trauma, burns, and/or exposure to bodies of water. Therefore, depending on the severity of the wound, surgical intervention may be necessary to prevent bacteremia and septic shock resulting from this bacterium. Symptoms of infection typically manifest 1 to 5 days after initial contact with the bacteria and may include fever, asthenia, adynamia, and other constitutional symptoms associated with infections [1, 2, 10, 12, 14, 16].

In the most recent studies on the new family *Shewanellaceae*, it has been noted that 40% of cases involve pure cultures of *Shewanella*, primarily associated with infections of the skin and soft tissues. These infections are often linked to skin breaks such as ulcers or trauma. Additionally, benign bacteremia has been described in only 2 studies, one of which was associated with severe hepatobiliary disease and malignancy, often with a fulminant course. However, the prevalent infections caused by *Shewanella spp*. in clinical samples are *S. putrefaciens* and *S. algae*, with over 80% of human isolates attributed to *S. algae*. Molecular methods based on 16 S rRNA and gryB are currently available to differentiate *S. algae* from *S. putrefaciens*. While primarily used in research laboratories, these two species can also be distinguished by their phenotypic characteristics and properties.

Both *S. algae* and *S. putrefaciens* are nonfermenting bacilli with a single polar flagellum, exhibiting growth on conventional solid media such as MacConkey agar, with colonies typically appearing yellow-brown and measuring 1–2 mm after 18–24 h of incubation. S. algae requires a temperature of 42 °C and a high concentration of NaCl (approximately 6%) for optimal growth. In comparison, *S. putrefaciens* is more saccharolytic, capable of fermenting maltose, glucose, and occasionally sucrose and arabinose [2, 11, 13, 14, 17–19].

In 2010, Gressier et al. described the first five cases of human spondylodiscitis caused by *S. algae*, suggesting that, like our patient, the portal of entry was likely a cutaneous lesion on the leg exposed to seawater. While cultures from vertebral disc biopsies identified *S. putrefaciens* using Automated Identification Systems, molecular identification revealed *S. algae*. This highlights the necessity of molecular typing to achieve precise bacteriological identification for appropriate antibiotic treatment [20].

The primary challenge encountered in typing the germ in secretion cultures is the isolation of oxidase-positive nonfermenting gram-negative bacilli, which can initially be mistaken for *Pseudomonas spp*. due to their similar bacterial morphology. However, *Shewanella spp*. exhibit a distinct innate resistance pattern compared to *Pseudomonas spp*., leading to the inappropriate use of antibiotics in these infections and the emergence of antimicrobial resistance. *S. algae* and *S. putrefaciens* are inherently susceptible to aminoglycosides, erythromycin, and quinolones, but resistant to penicillin and, in some cases, carbapenems. However, susceptibility to ampicillin and cephalosporins varies, with greater susceptibility to third and fourth generation cephalosporins compared to first and second generation cephalosporins [2, 3, 7, 11, 12, 14].

Currently, microbial resistance is on the rise, especially with the advent of genetic characterization. However, such characterization in *Shewanella* remains limited. The latest reported studies primarily characterize phenotypic resistance for clinical management purposes. These studies have identified genes encoding the β-lactamase of oxacillinase class D (blaOXA) conferring resistance to carbapenems, as well as *plasmid-mediated quinolone-resistance* (qnr) genes conferring resistance to quinolones. Additionally, intrinsic resistance to carbapenemases and quinolones has been established. In vitro studies have also demonstrated that the *S. oneidensis* Int1-like tyrosine recombinase can form antibiotic resistance integrons. Sulfonamide resistance-conferring genes sul1 and sul2 were detected in *S. algae* and *S. putrefaciens*, while the blaOXA-48-like gene was found in all three S. putrefaciens isolates. Genomic analyses have revealed that some *Shewanella spp*. can harbor between one and three plasmids ranging in size from 16 to 120 kb. Additionally, cryptic plasmids have been identified in certain *Shewanella* strains. Integrative and conjugative elements (ICE) similar to SXT/R391 have also been detected in *S. putrefaciens*. These elements have the capability to be excised from the host chromosome and self-transferred by conjugation [7].

## Conclusion

In recent years, there has been a rise in reported cases of *Shewanella* infections, primarily caused by S. algae and *S. putrefaciens*. This underscores the importance of considering *Shewanella* as a potential pathogen in patients with a clinical history indicative of exposure to bodies of water. Additionally, epidemiological surveillance is crucial for containing the spread of this pathogen, especially given the emergence of strains harboring antibiotic resistance genes that confer resistance to various antibiotics. Consequently, these resistance patterns must be taken into consideration when managing patients to prevent the development of intrinsic antimicrobial resistance. Further research is warranted to enhance our understanding of this genus, particularly its role in human infections.

## Data Availability

All data generated or analyzed during this study are included in this published article.

## Declarations

### Generative AI and AI-assisted technologies in the writing process

The authors declare that no AI or AI-assisted technologies were used in the research or writing process of this manuscript.
